# Carbon nanomaterials as promising substrates in the design of sensors for SARS-CoV-2 and new emerging viral infections

**DOI:** 10.2217/nnm-2021-0154

**Published:** 2021-08-25

**Authors:** Sepideh Abbaszadeh, Ghazal Nikaeen, Saeed Yousefinejad

**Affiliations:** ^1^Research Center for Health Sciences, Institute of Health, School of Health, Shiraz University of Medical Sciences, Shiraz, Iran

**Keywords:** biosensors, carbon nanomaterials, carbon nanotube, COVID-19, electrochemical, graphene, graphene oxide, immunosensor, optical nanosensor, SARS-CoV-2

The COVID-19 pandemic, induced by SARS coronavirus 2 (SARS-CoV-2), is obviously a hugely important health issue right now. Today, researchers all around the world are tirelessly working on how to combat COVID-19 in order to stop the spread of the disease. As no specific treatment is yet available, detection of the infected person at early stages of the disease is essential for better prognosis of COVID-19 [1]. Currently, conventional methods such as the real-time PCR technique are being carried out for detection of the coronavirus, but there are a number of challenges associated with this method. The detection process may take a few hours to a couple of days and, more importantly, requires expensive equipment or trained personnel for nasopharyngeal swab sample collection [2]. In addition, the application of these methods for rapid and inexpensive detection of the coronavirus is remarkably limited. Although swab sample collection may be self-administered, trained personnel are still required to run real-time PCR tests. It should also be taken into consideration that nucleic acid tests are more likely to produce false-negative results [3]. Therefore, there is an urgent need to develop rapid, ultrasensitive and cost-effective diagnostic tools that can identify people infected with COVID-19 at the early stages of the disease [4]. In recent years, studies have taken advantage of the superior properties of carbon-based nanomaterials, including their electrical and thermal conductivity [5] and high surface area [6], for the development of fast, selective and highly sensitive biosensors. This article provides an overview of graphene- and carbon nanotube (CNT)-based biosensing devices or based on other kind of carbon nanomaterials capable of detecting the COVID-19 virus.

## Recent applications of carbon nanomaterials in COVID-19 sensing

### Applications of graphene/graphene oxide in biosensors coupled with different analytical techniques

According to the importance of rapid tests in the sensing and diagnosis of COVID-19, different efforts have been made in the development of electrochemical sensors with fast responses. For example, Zhao *et al.* developed the first smartphone-based rapid and accurate sensor for electrochemical determination of SARS-CoV-2 without RNA amplification by developing a supersandwich electrochemical biosensor based on SCX8 functionalized reduced graphene oxide (GO) [7]. The detection limit of this biosensor was confirmed to be 200 copies/ml in the clinical specimen. With regard to the development of such sensors, GO possesses a suitable surface for grafting sensing agents and enhancing the surface of sensing media for the purposes of combining other metal or metal oxide nanomaterials to amplify selectivity or sensitivity. This approach can be performed without the need for current lab-based nucleic acid amplification and reverse transcription. This assay also proved to have the potential to be used in the future for point-of-care testing. Next, the researchers tested the response to other respiratory pathogens, such as SARS-CoV-1, Middle East respiratory syndrome coronavirus, human coronavirus OC43 and influenza A, and it was found that their biosensing platform showed no electrochemical response, establishing that their sensor was specific for SARS-CoV-2. Unlike quantitative real-time PCR, this kind of SARS-CoV-2 biosensor can provide detection of low viral specimens from the upper respiratory tract, feces, urine and plasma.

As another application of graphene-based materials, Seo *et al.* have developed a rapid graphene-based field-effect transistor biosensor for the detection of SARS-CoV-2 by coating a graphene sheet with the viral spike protein antibody [8]. It was reported that the graphene-based field-effect transistor biosensor provided low noise and ultrasensitive determination by detecting changes in the area surrounding the surface of the graphene sheet. This field-effect transistor device was able to detect the SARS-CoV-2 spike protein in human nasopharyngeal swab specimens. Limits of detection of 1.6 × 10^1^ plaque-forming units/ml and 2.42 × 10^2^ copies/ml were obtained in culture media and clinical samples, respectively. As expected, based on this work and previously reported studies, in the near future, graphene-based biosensors will be able to take advantage of viral antibodies applied to graphene sheets in the development of promising ways to rapidly sense new emerging viruses [9,10].

Ali *et al.* developed a biosensor for detecting the receptor binding domain (RBD) of SARS-CoV-2 and the S1 subunit of its spike protein in biological samples [11]. The biosensor has 3D electrodes created by 3D nanoprinting and coated with nanoflakes of reduced GO, which are immobilized with the specific viral antigens. The electrode was used in a standard electrochemical cell after connecting to a microfluidic device. With the use of impedance spectroscopy, it was noted that the impedance of the electrical circuit changed when specific antigens were introduced on the surface of the electrode. The detection limits of the proposed nanosensor were 2.8 and 16.9 × 10^-^^15^ M for the spike protein and RBD, respectively. This study was developed based on the latest advances in materials and manufacturing methods, including nanoparticle 3D printing, to detect antibodies specific to SARS-CoV-2 within seconds. Interestingly, it should be noted that this biosensing platform could be regenerated within a minute for reuse by eluting the antibodies from the antigens with a low-pH solution consisting of 1.0 m (pH 2.5) formic acid solution. After some consideration to making it suitable for routine usage, this can be an advantage with regard to widespread application.

GO again showed its benefits in an Au/fiber Bragg grating sensor decorated with GO, which was developed by Samavati *et al.* for detecting SARS-CoV-2 in the saliva of patients in different phases of the disease [12]. The sensor measures the association between virus density and changes in the sensing elements, and the disease level is identified by the deviation in observed light wavelength in suspect samples compared with healthy samples. The fabricated probe combines the advantages of GO and gold nanomaterials for accurate, sensitive and reliable detection of the virus. In their research, the Au nanolayer was deposited on the fiber Bragg grating sensor. Next, the sensing part was decorated with GO. Although the combination of GO and gold nanomaterials was applied here as the first sensing nanoprobe for SARS-CoV-2 virus detection, application of other metals or metal composites can be evaluated for viral sensing in future studies.

Mojsoska *et al.* reported a novel immunosensor composed of a printed graphene layer as a working electrode functionalized with PBSE as a linker bound to a monoclonal antispike antibody [13]. After binding the antigen with the incubated sensor, an absolute change in ferri/ferrocyanide current was produced, which was utilized to detect the spike protein. This sensor detected a specific signal of the S1 subunit of the recombinant spike protein above 260 nM (20 μg/ml) and also detected SARS-CoV-2 at a concentration of 5.5 × 10^5^ plaque-forming units/ml.

Recently, Torrente-Rodríguez *et al.* developed a novel multiplex, high-performance immunosensor using laser-engraved graphene working electrodes for ultrasensitive rapid electrochemical detection of SARS-CoV-2 [14]. This novel platform, known as SARS-CoV-2 RapidPlex, can be used efficiently at the point of care. The mechanism is based on the detection of N protein; specific antibodies, including immunoglobulin M and immunoglobulin G, of SARS-CoV-2; and C-reactive protein levels in blood and saliva samples as an indicator of the severity of the disease. In addition, to ensure the applicability of the system, the RapidPlex platform was evaluated using blood and saliva samples that were either positive or negative for COVID-19. The laser-engraved graphene-based RapidPlex device proved to be a fast, sensitive and easy-to-use system for determination of COVID-19 in patients. It is important to note that the advantageous characteristics of graphene, such as its high charge mobility and large surface area, enabled this system to reliably diagnose COVID-19 in a telemedicine setting with an incubation time of just 1 min.

Hashemi *et al.* designed a nanosensor composed of working electrodes coated with a layer of decorated GO, sensitive chemical compounds and Au nanostars to detect target viruses in various biological samples [15]. To ensure the sensitivity of the nanosensor for coronavirus, 100 blind nasopharyngeal swab samples were tested using the differential pulse voltammetry technique. It was demonstrated that the differential pulse voltammetry pattern of SARS-CoV-2 appeared at a different position from other types of viruses and their strains, proving that this nanosensor could be a rapid and specific nanosensor for the detection of infected people at any stage of the disease.

### Applications of nanocarbon black-based materials

Screen-printed electrodes, which make use of the benefits of carbon nanomaterials, are another type of electrochemical immunoassay employed in the detection of SARS-CoV-2. Fabiani *et al.* developed an electrochemical immunoassay and reported the smart and rapid detection of the spike and nucleocapsid proteins of SARS-CoV-2 in saliva samples [16]. Their magnetic bead-based electrochemical assay worked by immobilizing antibodies for the spike and nucleocapsid proteins and then reading them with screen-printed electrodes modified with carbon black nanomaterials combined with a portable potentiostat. The assay's limits of detection for the spike and nucleocapsid proteins in untreated saliva samples were 19 and 8 ng/ml, respectively.

### Applications of CNT-based materials

In addition to graphene and GO, the unique benefits of other kinds of carbon nanomaterials that have been applied in previous nano/biosensors cannot be ignored in the field of viral detection. Pinals *et al.* developed an accessible, rapid nanosensor for the detection of COVID-19 via the conjugation of single-walled CNTs with an entry receptor of the SARS-CoV-2 spike protein (angiotensin-converting enzyme 2) [17]. The researchers used single-walled CNTs because of their superior application in biological analyte sensing and intrinsic near-IR fluorescence. Also, it was found that a 90-min exposure between the nanosensor and SARS-CoV-2 spike protein provided a twofold increase in nanosensor fluorescence activity. The limit of detection of the nanosensor was 9.5 nM spike RBD in viral transport medium and saliva. Thus, the use of CNTs in the development of spectroscopic sensors for enhancing the fluorescence properties of biological reagents can be an interesting subject in the detection of SARS-CoV-2 or similar emerging viral infections.

CNTs have also been utilized in electrochemical sensors for the detection of SARS-CoV-2. An electrochemical sensor fabricated with multiwalled CNTs on the tips of steel needles was developed by Miripour *et al.* to quantitate levels of reactive oxygen species induced by viral activity of SARS-CoV-2 in sputum [18]. The applicability of the sensor was evaluated in more than four hospitals and proved to be effective in the rapid identification of COVID-19 in patients.

Badhe *et al.* developed two biosensing platforms based on a graphene sheet and a CNT functionalized with two screened peptides that were effective for SARS-CoV-2 detection [19]. Detection of SARS-CoV-2 was provided through rapid binding of the RBD antigen of the virus to the proposed peptides. These platforms were evaluated in both *in vivo* and *in vitro* experiments.

As another interesting example of the manufacture of spectroscopic sensors for SARS-CoV-2 using CNTs, Jadhav *et al.* proposed a diagnostic tool for rapid screening of individuals with symptomatic and asymptomatic COVID-19 based on the surface-enhanced Raman spectroscopy technique [20]. In this study, two models were reported: one with a disposable electrospun micro/nanofilter for the rapid detection of asymptomatic COVID-19 patients and one with a vertically aligned CNT functionalized microchannel for symptomatic COVID-19 patients. This platform proved to have the great potential of not only being used to detect COVID-19 but also being useful for the rapid detection or quantitation of other emerging viral infections and helping to control future pandemics.

## Conclusion

Early diagnosis of the disease can be highly beneficial in how we treat COVID-19 patients. SARS-CoV-2 has had a devastating effect around the world. Studies have demonstrated that the application of CNTs, functionalized or in combination with other materials, can help improve their properties and allow the development of simple, highly sensitive, mass-produced biosensors for use in not only the COVID-19 pandemic but also future pandemics.

There are currently various methods for measuring coronaviruses in biofluid, but because the virus is highly contagious and infected individuals need to be identified as quickly as possible, many studies have been performed to develop newer methods with reduced response time, greater cost–effectiveness and more sensitivity. Of the tested methods, carbon nanoparticles are very important because of their high surface area for reacting to and bonding with other sensing moieties. However, these methods still cannot be introduced as the main alternative to the PCR test, which is the gold standard test. The promising results with regard to the application of different kinds of carbon nanomaterials in designing sensors for new emerging viruses such as SARS-CoV-2 have been reviewed and discussed. Based on these results, more modifications and applications of CNTs and graphene in combination with other nanomaterials are expected with the introduction of accurate and rapid tests for new viral infections in the near future. Reports published in the last year show the benefit of carbon nanomaterials such as graphene, GO and single/multiwalled CNTs in SARS-CoV-2 sensing with more applications in electrochemical studies compared to other analytical methods, such as spectroscopy or separation based methods. However, the performance of these carbon materials in spectroscopic sensors cannot be ignored. The experience in this field has the potential to address challenges associated with rapid and accurate diagnosis of new emerging viral infections.
